# pH-Responsive Collagen Hydrogels Prepared by UV Irradiation in the Presence of Riboflavin

**DOI:** 10.3390/ijms251910439

**Published:** 2024-09-27

**Authors:** Shoki Setoyama, Ryota Haraguchi, Shigehisa Aoki, Yushi Oishi, Takayuki Narita

**Affiliations:** 1 Department of Chemistry and Applied Chemistry, Saga University, Saga 840-8502, Japan; 2 Department of Pathology and Microbiology, Saga University, Saga 849-8501, Japan

**Keywords:** collagen, hydrogel, UV crosslinking, pH-responsive, volume phase transition

## Abstract

This study reveals the pH-responsive behavior of collagen hydrogels prepared using ultraviolet (UV) irradiation with riboflavin as a photosensitizer. By varying the UV exposure time, we modulated the crosslinking density, thereby influencing the mechanical properties and pH responsiveness. Rheological analysis confirmed successful network formation, whereas swelling studies revealed significant pH-dependent behavior, with maximum swelling at a pH of four and minimal swelling above a pH of six, demonstrating partial reversibility over multiple pH cycles. Mechanical testing showed a pH-dependent elastic modulus, which increased 10 fold from a pH of 6 to 10. Fibroblast proliferation assays confirmed the biocompatibility of the hydrogels, with cell growth positively correlating with the UV exposure time. This research demonstrates the potential of UV-crosslinked collagen hydrogels in biomedical applications, such as tissue engineering and drug delivery, where pH responsiveness is essential.

## 1. Introduction

Owing to their ability to undergo volumetric phase transitions in response to changes in pH levels, pH-responsive hydrogels have garnered significant attention [1,2,3,4]. These materials have potential applications in various biomedical fields, including drug delivery, tissue engineering, and wound healing. However, these hydrogels do not always exhibit optimal biocompatibility, which is a critical factor for their application in various biomedical fields [5]. Synthetic hydrogels in particular often lack biocompatibility, and concerns have been raised regarding the potential toxicity associated with their degradation byproducts [5]. These limitations have motivated researchers to develop new hydrogels with improved biocompatibility. Recent research has focused on designing pH-responsive hydrogels which not only respond to pH changes but also possess enhanced biocompatibility [6,7,8,9]. For instance, hydrogels composed of polypeptides [6] and chitosan-based hydrogels [10] have been identified as promising pH-responsive biomaterials, owing to their excellent biocompatibility and nontoxicity. These innovations, driven by the use of biocompatible polymers and advanced synthesis techniques, have expanded the potential applications of pH-responsive hydrogels in the biomedical field [11,12].

Collagen, the most abundant natural protein in the mammalian body, has attracted significant attention in tissue engineering [13,14,15], wound healing [16,17], drug delivery [18,19,20], and cosmetics [21,22,23] due to its unique triple-helix structure and high biocompatibility [24,25]. Its biodegradability and stability under physiological conditions make collagen a promising material for regenerative medicine applications [26,27]. The triple-helical structure of collagen provides cell adhesion sites which promote cell proliferation and differentiation, making it an excellent scaffold for tissue regeneration [28,29,30]. Furthermore, the high biocompatibility of collagen ensures its safety in clinical applications, particularly when compared with many synthetic materials [31,32,33].

The pH-dependent solubility of collagen in water [34,35] has been used to develop pH-responsive collagen dressings which can adapt to wound environments, promote faster healing, and improve infection control [36,37]. These dressings can adjust their properties according to the pH of the wound, promoting faster healing and improving infection control through the pH-triggered release of antimicrobial agents. Despite its high functionality, collagen alone has low mechanical strength, which limits its use [7]. To overcome this limitation, various chemical methods have been employed to transform collagen into hydrogels, thereby improving their mechanical properties and stability [38,39,40,41]. This approach has facilitated the development of devices suitable for long-term in vivo use with controlled degradation [42,43,44], opening up possibilities as cell scaffolds beyond what can be achieved with solution systems.

However, glutaraldehyde, a commonly used chemical crosslinking agent, is unsuitable for medical applications, owing to its cytotoxicity [45,46,47]. Additionally, there are few reports on the pH response of gelatinized collagen gels by chemical crosslinking. This is likely because collagen gels formed using chemical crosslinkers utilize amino or carboxyl groups, the primary sources of pH responsiveness for collagen, as crosslinking sites [48,49,50,51], thereby diminishing their pH sensitivity. On the other hand, the pH responsiveness of collagen gels formed using gamma irradiation has been reported, but this method requires specialized radiation sources such as cobalt-60, limiting its general applicability. Furthermore, these gels tend to dissolve at pH levels below three.

In this study, we focused on the pH responsiveness of hydrogels obtained by UV irradiation of collagen solutions containing riboflavin [38,52]. This method promotes crosslinking between collagen molecules, resulting in the formation of stable hydrogels. Upon UV irradiation, riboflavin acts as a photosensitizer, generating reactive oxygen species which facilitate collagen fiber crosslinking, thereby enhancing the mechanical properties and stability of the biomaterial [53,54]. This method is superior to chemical crosslinking methods in terms of biocompatibility because it avoids associated toxicity issues [55,56]. Moreover, it does not require specialized equipment and offers a more accessible and straightforward gelation process. An additional advantage of UV irradiation is its potential to enhance the pH responsiveness of the gels. This hypothesis is based on the observation that UV-crosslinked gelatin nanogels exhibit greater temperature sensitivity than those crosslinked with glutaraldehyde [57].

To investigate the pH responsiveness of collagen gels crosslinked by photoactivation of riboflavin, the following evaluations were conducted. (1) The gelation process was characterized by dynamic viscoelastic analysis of collagen solutions containing riboflavin when irradiated with UV light and the effects of UV light irradiation on the rheological and mechanical properties of the collagen solutions. (2) We evaluated the pH-induced volume phase transition of the hydrogel by measuring its swelling over a wide pH range (4–10) and evaluating the reversibility of this reaction through multiple pH cycles. (3) The relationship between the pH and mechanical properties of the hydrogel was investigated by measuring the elasticity at various pH values. (4) The biocompatibility and potential of these hydrogels as cell scaffolds were evaluated by assessing the proliferation of fibroblasts in hydrogels prepared using varying amounts of UV irradiation.

Although the preparation of collagen hydrogels using UV irradiation with riboflavin as a photosensitizer has been previously reported [52,55], the pH-responsive behavior of these hydrogels has not been thoroughly investigated. Our study builds upon these earlier works by focusing on the pH-dependent properties of UV-crosslinked collagen hydrogels, exploring their swelling behavior, mechanical properties, and biocompatibility across a range of pH values. This study provides new insights into the potential applications of these materials in pH-sensitive biomedical contexts.

By addressing these aspects, we aimed to provide a comprehensive understanding of UV-crosslinked pH-responsive collagen hydrogels and their potential applications in tissue engineering, drug delivery, and wound healing. This study advances smart biomaterials which dynamically respond to physiological changes, potentially enhancing biomedical interventions. The approach presented here offers a simple, efficient, and scalable method for producing pH-responsive collagen hydrogels, overcoming the limitations of conventional techniques and paving the way for advanced biomaterial design.

## 2. Results and Discussion

### 2.1. UV-Induced Gelation of Collagen Solutions

**Figure 1:**
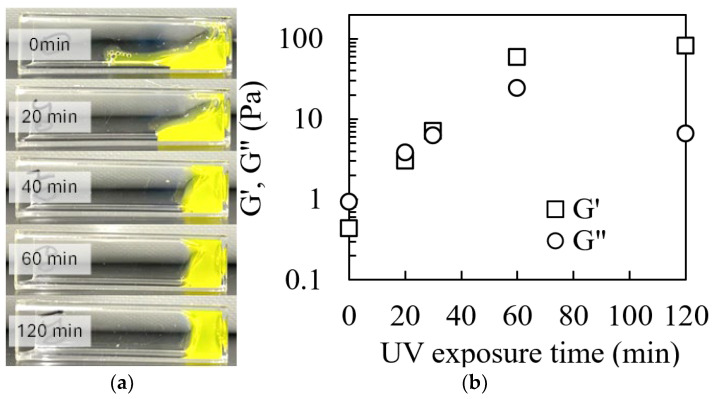
Rheological properties of riboflavin-mixed collagen solutions before and after UV irradiation. (**a**) Visual demonstration of the flow behavior in collagen solutions exposed to varying UV irradiation times. Images were captured 30 s after tilting the sample containers. (**b**) Storage modulus (G′) and loss modulus (G″) as a function of UV exposure time.

The effects of UV irradiation on the rheological properties of collagen solutions containing 0.02 mM riboflavin were investigated. Figure 1a shows the deformability of 1% (*w*/*v*) collagen solution subjected to UV irradiation for 0, 20, 40, 60, and 120 min. The non-irradiated collagen solution exhibited a liquid-like behavior, flowing immediately upon tilting the container. In contrast, the UV-irradiated solutions displayed a transition to semi-solid-like behavior with increasing resistance to flow as the irradiation time increased. Figure 1b illustrates the changes in the viscoelastic properties of the samples prepared with different UV irradiation times. Both the loss modulus (G″) and storage modulus (G′) increased approximately linearly with the UV irradiation time. The increase in G′ and G″ upon UV irradiation indicated progressive stiffening of the collagen network. Notably, G′ surpassed G″ after 40 min of UV irradiation, indicating the onset of gelation. This crossover point (G′ = G″) marks the transition from the liquid to the gel state, with further irradiation reinforcing the gel structure. The observed increase in the G′ and G″ values post UV irradiation can be attributed to an increase in molecular weight resulting from photocrosslinking [58,59]. This phenomenon elucidates the transformation of collagen solutions from a liquid state to a gel-like state. UV-induced crosslinking, mediated by riboflavin as a photosensitizer, likely promotes the formation of intermolecular covalent bonds between collagen molecules, thereby enhancing the structural integrity and mechanical properties of the resulting hydrogel. These findings are consistent with those of previous studies on UV-crosslinked collagen systems [55,56].

While this study primarily focused on the pH-responsive behavior of UV-crosslinked collagen hydrogels, it is worth briefly discussing the potential crosslinking mechanism. Based on previous studies on riboflavin-mediated UV crosslinking of collagen [60,61], we hypothesized that crosslinking occurs primarily between amino acid residues and is catalyzed by the generation of singlet oxygen. This process likely favors carbonyl-based crosslinking reactions. Importantly, it has been suggested that free amine groups do not play a major role in the crosslinking process [54]. This characteristic is crucial for maintaining the pH responsiveness of our hydrogels, as the amine groups remain available for protonation and deprotonation in response to pH changes. However, it should be noted that a quantitative analysis of the specific crosslinking structures formed in our system is beyond the scope of this study and would require further investigation using advanced spectroscopic techniques.

### 2.2. pH-Responsive Swelling Behavior of UV-Crosslinked Collagen Hydrogels

The pH-dependent swelling behavior of UV-irradiated crosslinked collagen hydrogels was investigated over a wide pH range (3–10). At pH ≤ 3, the collagen gel swelled to such an extent that it could not be manipulated using tweezers, precluding quantitative swelling measurements. However, at pH 4–10, the gel maintained sufficient structural integrity for handling and shape retention.

**Figure 2:**
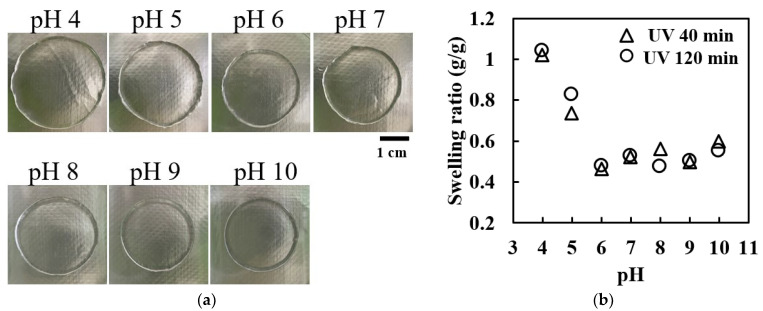
The pH-dependent swelling of UV-irradiated collagen hydrogels: (**a**) representative photographs of collagen gel disks equilibrated in buffers of varying pH levels (UV irradiation time of 40 min) and (**b**) swelling ratios of collagen gel disks as a function of the buffer pH for two UV irradiation times (40 and 120 min).

Figure 2a displays photographs of the collagen gel disks (prepared with 40 min of UV irradiation) equilibrated in buffer solutions ranging from a pH of 3 to 10. These images clearly demonstrate that the hydrogel exhibited a larger diameter at a pH of four and a smaller diameter at a pH ≥6. Figure 2b shows the swelling ratio as a function of the pH, revealing significant swelling in buffers with a pH ≤6, particularly at a pH of four. In this range, the swelling ratio exhibited a negative linear relationship with the pH. Above a pH of six, the swelling ratio stabilized at a relatively low value of approximately 0.5.

This pH-dependent swelling behavior closely mirrors the pH-dependent solubility of the collagen molecules. The observed decrease in swelling at a pH > 6 corresponds to the known increase in turbidity in collagen solutions in this pH range [59]. Notably, the gels crosslinked for 40 and 120 min showed nearly identical pH-dependent swelling trends, indicating that UV irradiation did not impair the pH sensitivity of the gel. This preservation of the pH sensitivity suggests the retention of ionic groups within the gel structure post UV crosslinking.

Interestingly, the gels formed by 120 min of UV irradiation exhibited approximately 1.5 times smaller gel weights (W_1_ and W_2_) than those formed by 40 min of irradiation. The swelling behavior of polyelectrolyte gels is governed by the balance between the elastic shrinkage force of the gel chains (determined by the Donnan osmotic pressure) and the gel density (determined by the concentration of ionic groups) [62,63,64]. Assuming that UV crosslinking does not alter the concentration of ionic groups, the decrease in gel weight with increasing UV irradiation doses suggests an increase in the crosslinking density within the gel.

Importantly, the UV crosslinking method appeared to preserve these ionizable groups, as evidenced by the maintained pH responsiveness of the hydrogels. This is in contrast with some chemical crosslinking methods which may consume these groups during the crosslinking process, potentially reducing the pH sensitivity of the resulting hydrogels. In contrast to gamma-irradiated collagen gels, which are known to dissolve at a pH ≤ 5, our UV-irradiated gels showed no signs of degradation or dissolution at a pH of four even after 120 min of UV irradiation. This stability at a low pH presents a significant advantage for applications in acidic environments. The enhanced stability of UV-crosslinked gels compared with gamma-irradiated gels may be attributed to different crosslinking mechanisms. Although high-energy gamma radiation can cause both crosslinking and degradation to compete, often resulting in brittle gels [65,66], UV irradiation in the presence of riboflavin appears to promote more controlled crosslinking without significant degradation.

Because the pH responsiveness of UV-crosslinked collagen hydrogels closely resembles the pH dependence of the turbidity of collagen solutions, the pH-responsive behavior of this gel should also be due to the presence of various ionizable groups along the collagen molecules. These include carboxyl groups (–COOH) from aspartic and glutamic acids, as well as amino groups (–NH_2_) from lysine and arginine. At low pH values (pH < 6), the amino groups became protonated (–NH_3_^+^), increasing the overall positive charge of the hydrogel. This led to electrostatic repulsion between the chains and an increased Donnan osmotic pressure, causing the gel to swell. Conversely, at higher pH values (pH > 6), the carboxyl groups became deprotonated (–COO^-^), increasing the negative charge, and the screening effect of the charge reduced the Donnan osmotic pressure, causing the gel to shrink. The isoelectric point of collagen (approximate pH of 6–7) represents the point of minimal net charge, corresponding to the observed minimal swelling in our hydrogels. This behavior is consistent with the pH-induced volume phase transitions observed in other polyelectrolyte hydrogels, such as crosslinked chitosan [67,68]. To confirm whether the pH-responsive change in the swelling degree shown here was a true volume phase transition phenomenon, we investigated the reversibility of the swelling response to a pH change in the next section. This investigation is also important for understanding the potential of these hydrogels in applications which require repeated pH-responsive behaviors, such as controlled drug delivery systems and smart wound dressings.

### 2.3. Reversibility of pH-Responsive Behavior in UV-Crosslinked Collagen Hydrogels

**Figure 3:**
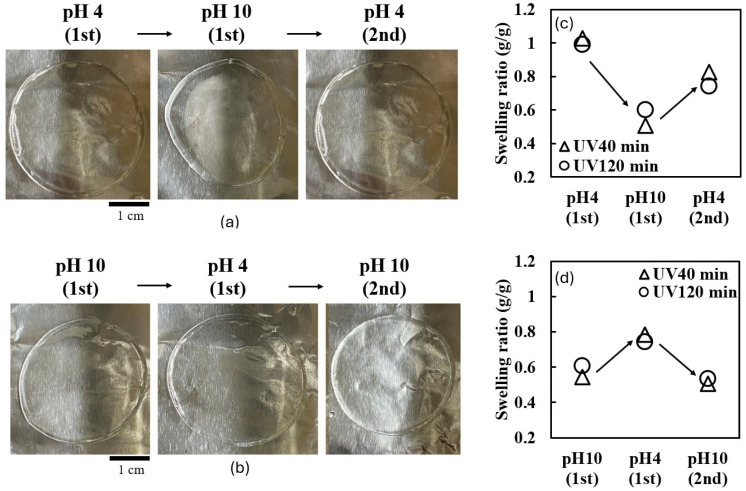
Reversible pH-responsive behavior of UV-crosslinked collagen hydrogels. (**a**) Representative photographs of collagen gel disks (40 min of UV irradiation) cycled through pH 4 → pH 10 → pH 4. (**b**) Representative photographs of collagen gel disks (40 min of UV irradiation) cycled through pH 10 → pH 4 → pH 10. (**c**) Swelling ratios of hydrogels during pH 4 → pH 10 → pH 4 cycle. (**d**) Swelling ratios of hydrogels during pH 10 → pH 4 → pH 10 cycle.

The reversibility of pH-responsive behavior in UV-crosslinked collagen hydrogels was investigated through cyclic pH changes. Figure 3 illustrates this behavior for the hydrogels prepared with 40 and 120 min of UV irradiation.

Figure 3a,b shows the morphological changes of hydrogels cycled through pH 4 → 10 → 4 and pH 10 → 4 → 10, respectively. In both cases, the hydrogels demonstrated a degree of reversibility, nearly returning to their original size when reimmersed in the initial pH buffer.

A quantitative analysis of the swelling behavior is presented in Figure 3c,d. For the pH 4 → 10 → 4 cycle (Figure 3c), both the 40 min and 120 min irradiated gels showed incomplete recovery upon returning to a pH of four. The 40 min irradiated gel reached a swelling ratio of 0.85, while the 120 min irradiated gel only recovered to 0.7.

For the pH 10 → 4 → 10 cycle (Figure 3d), the 40 min irradiated gel achieved a swelling ratio of 0.8 at a pH of four, while the 120 min irradiated gel only reached 0.7. Upon returning to a pH of 10, the 120 min irradiated gel recovered to a swelling ratio of 0.75.

These results demonstrate partially reversible pH-responsive behavior, with some hysteresis observed, particularly after exposure to a pH of 10. The incomplete recovery of the swelling ratio, which was especially pronounced in the gels irradiated for 120 min, suggests irreversible changes in the hydrogel structure during pH cycling.

This behavior can be attributed to the structural changes in collagen at high pH levels. Previous studies have shown that collagen can denature at a pH of 10, leading to reduced gelling properties and water-holding capacity [69,70]. The more significant loss of water-holding capacity in gels irradiated for 120 min suggests that longer UV exposure may result in a more rigid network structure, which is more susceptible to irreversible changes upon pH cycling.

This observed partial reversibility has important implications for potential applications. While these hydrogels demonstrate the ability to respond to pH changes multiple times, the gradual loss of swelling capacity, particularly after exposure to high pH levels, must be considered in their design for specific uses. Future studies should focus on enhancing the reversibility of the pH response, possibly through the incorporation of additional stabilizing agents or optimization of the crosslinking process.

### 2.4. Mechanical Properties of UV-Crosslinked Collagen Hydrogels at Various pH Levels

**Figure 4:**
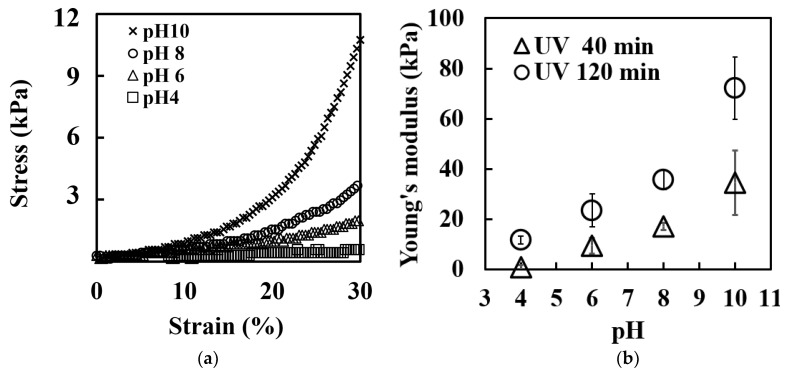
Mechanical properties of UV-crosslinked collagen hydrogels at varying pH levels. (**a**) Stress–strain curves from compression tests on hydrogels (40 min UV irradiation) equilibrated in different pH buffers. (**b**) Young’s modulus of hydrogels calculated from the linear regions of stress-strain curves.

The mechanical properties of the UV-crosslinked collagen hydrogels were investigated in different pH environments to further elucidate their pH-responsive behavior. Figure 4a presents the stress–strain curves obtained from the compressive tests on gels immersed in various pH buffer solutions. The curves demonstrate that higher pH values corresponded to steeper slopes, indicating increased stiffness.

Figure 4b quantifies this trend by showing the Young’s modulus, derived from the linear portion of the stress–strain curves. Notably, the elastic modulus of the gel immersed in the buffer with a pH of 6 was approximately 10 times lower than that of the gel immersed in the buffer with a pH of 10. This trend was consistent across different UV irradiation times, supporting the pH-responsive nature of the mechanical properties of these hydrogels in the pH range of 6–10.

The observed increase in both tensile strength and elastic modulus from neutral to alkaline pH levels aligns with the properties of conventional acidic collagen gels which are physically crosslinked [71,72]. This similarity leads to two key conclusions. (1) UV-induced crosslinking preserves the pH-dependent characteristics of collagen, and (2) the increased elastic modulus at high a pH level may suppress swelling deformation, resulting in similar swelling behavior (shrinkage state) at pH levels of 6 and 10.

Interestingly, the pH dependence of the swelling ratio showed a minimal change between a pH of 6 and 10 (Section 2.2), which appears to contradict the general understanding that the elastic modulus increases with the pH while the swelling ratio remains constant and that hydrogels with higher water contents typically exhibit lower elastic moduli [73]. This apparent contradiction can be resolved by considering that the high elastic modulus at a pH of 10 suppresses gel deformation, resulting in a swelling degree at a pH of 10 which is similar to that at a pH of 6. If the elastic modulus is low near a pH of 10 (near the isoelectric point of acid-soluble collagen (pH of 6–7)), the increased Donnan osmotic pressure due to carboxyl group dissociation would likely lead to higher swelling, but this is counteracted by the increased elastic modulus.

The increase in the elastic modulus at higher pH values can be attributed to several factors. As the pH increases, the net positive charge decreases, owing to deprotonation of the collagen monomer side chains, which improves the hydrogen bonding capacity between the collagen triple helices [74]. This enhanced hydrogen bonding not only stabilizes the collagen structure but also promotes fibril formation, resulting in a more compact and stable fibril arrangement [71,72]. The presence of hydrogen bonds strengthens the interactions between collagen fibrils, leading to a more cohesive and mechanically stable gel [75,76].

Notably, the gels prepared with 120 min of UV irradiation exhibited approximately twice the elastic modulus of those prepared with 40 min of irradiation. This result suggests that prolonged UV crosslinking may promote hydrogen bonding or increase the crosslinking density through the formation of additional chemical crosslinks, although further investigation is needed to confirm these mechanisms.

These findings highlight the complex interplay between the pH, swelling behavior, and mechanical properties of UV-crosslinked collagen hydrogels. The ability to modulate the mechanical properties of these gels by changing the pH while maintaining a relatively constant swelling ratio at higher pH values presents intriguing possibilities for applications requiring precise control of the material stiffness without significant volume changes.

### 2.5. Cell Affinity and Proliferation on UV-Crosslinked Collagen Hydrogels

**Figure 5:**
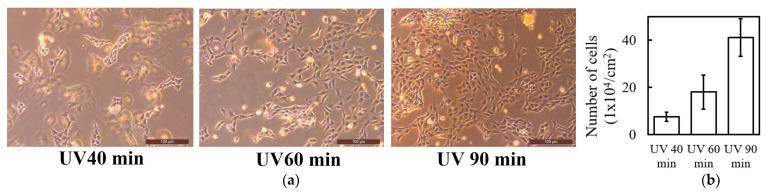
(**a**) Microscopic images and (**b**) quantitative analysis of fibroblast proliferation on UV-crosslinked collagen hydrogels prepared with different UV irradiation times (40, 60, and 90 min) on day 2 of culture.

To evaluate the cell affinity of the UV-crosslinked collagen hydrogels, we investigated fibroblast proliferation on gels prepared with varying UV irradiation times (40, 60, and 90 min). Figure 5a shows microscopic images of the fibroblasts cultured on gels on the second day of culture. Microscopic images of fibroblasts immediately after seeding (before culture) on UV-crosslinked collagen hydrogels prepared with different UV irradiation times are shown in Supplementary Figure S1. No significant differences in cell density or morphology were observed immediately after seeding. Fibroblasts were evenly distributed across the collagen gels regardless of the crosslinking time, suggesting that initial cell adhesion was not substantially affected by UV irradiation duration. However, significant differences were observed on the second day of culture. A comparison with the initial cell distribution (Supplementary Figure S1) demonstrated a significant increase in cell density and morphological changes over the culture period. The cell density increased with the UV irradiation time, indicating enhanced cell proliferation. Fibroblasts exhibit characteristic spindle shapes and form interconnected networks, suggesting a favorable environment for cell proliferation and intercellular communication.

Figure 5b shows the quantification of the cell numbers on day 2 for each UV irradiation condition. Consistent with microscopic observations, the initial cell numbers were similar across all conditions (approximately 1.8–2.2 × 10^4^ cells/cm^2^), with no statistically significant differences. This further confirms that the UV crosslinking duration did not significantly affect the initial cell adhesion. The cell density data on day 2 revealed a clear correlation between the UV irradiation time and cell proliferation. The 90 min irradiated gel exhibited the highest cell density, reaching approximately 40 × 10^4^ cells/cm^2^. While cell numbers increased in gels with shorter UV exposure times, the extent of proliferation was lower than that in the 90 min exposure gel.

These results indicate a relationship between the UV exposure time, which directly affected the degree of collagen crosslinking, and the capacity of the gel to support cell adhesion and proliferation. This observation aligns with previous studies on UV-crosslinked collagen gels using riboflavin as a photosensitizer, which have shown effective proliferation of human corneal epithelial cells (HCECs) [55]. The stabilizing effect of UV irradiation on collagen fibrils is thought to enhance cell spreading and improve adhesion properties compared with non-irradiated collagen. Fibroblasts cultured on photocrosslinked collagen gels have been reported to exhibit a myofibroblast phenotype, associated with enhanced cell functions, such as contraction and migration [77]. Cells cultured in scaffolds with optimal UV crosslinking often exhibit a spread-out, elongated morphology, leading to enhanced cell function [78].

The observed enhancement in fibroblast proliferation with increasing UV irradiation times can be attributed to several factors. First, UV crosslinking, unlike chemical crosslinking methods, forms bonds primarily between aromatic residues such as tyrosine and phenylalanine [79,80]. This process maintains the integrity of the integrin-binding sites which are crucial for fibroblast adhesion and proliferation. The preservation of these sites likely contributed to the observed favorable cell behavior across all UV-crosslinked samples. Second, longer UV irradiation times resulted in an increased crosslinking density, leading to enhanced mechanical properties for the hydrogels [77]. As observed in Section 2.4, the gels prepared with longer UV irradiation times exhibited higher elastic moduli. This increase in stiffness may provide a more favorable mechanical environment for fibroblast growth because cells are known to respond to the mechanical properties of their substrates. Furthermore, UV crosslinking improves the stability of collagen fibrils, which may contribute to better cell spreading and improved adhesion properties compared with non-irradiated collagen [79,81]. This stabilization effect may explain the progressive increase in cell proliferation observed with longer UV irradiation times. The crosslinking process may also alter the surface structure of the gel, creating a topography which promotes cell attachment and proliferation. Although not directly measured in this study, changes in surface characteristics could be a contributing factor to the observed cell behavior.

However, it is important to note that although longer UV irradiation times resulted in improved cell proliferation in this study, there may be an upper limit to this effect. Excessive crosslinking could potentially lead to over-stiffening of the hydrogel or alterations in the collagen structure, which might negatively affect the cell behavior. Future studies should focus on determining the optimal range of UV crosslinking for various cell types and investigating the long-term effects of these materials on cell behavior and tissue formation.

Further research is needed to elucidate the specific mechanisms by which UV crosslinking enhances cell proliferation. This could include a detailed analysis of the hydrogel microstructure, quantification of available cell adhesion sites, and investigation of cell signaling pathways activated in response to the crosslinked collagen environment.

## 3. Materials and Methods

### 3.1. Materials

A native collagen acidic solution (IAC-50, 5 mg/mL) was purchased from Koken (Tokyo, Japan). The Good’s buffers used were Tricine, TES, CHES, HEPES, and MES. The pH buffers were obtained from Dojindo Laboratories (Kumamoto, Japan). Sodium hydroxide and riboflavin were purchased from Wako Pure Chemical Industries (Osaka, Japan). All chemicals and reagents were of analytical grade and were used without further purification. Ultrapure water (Milli-Q, Millipore, Billerica, MA, USA) was used for all experiments.

### 3.2. Preparation of Collagen Hydrogels

A 0.02 mM riboflavin-mixed collagen solution was prepared by combining the commercial collagen solution with riboflavin in a beaker. The mixture was stirred at 7 °C for 12 h using a magnetic stirrer (500 rpm) and then cooled to 4 °C for stabilization prior to UV irradiation. Collagen gels were fabricated via UV-initiated crosslinking. Aliquots of the riboflavin-mixed collagen solution were carefully pipetted into circular depressions (20 mm in diameter and 2 mm deep) on a polytetrafluoroethylene (PTFE) plate. The samples were then subjected to UV irradiation (λ = 366 nm) for either 40 or 120 min using a UV lamp (CAMAG^®^ UV Lamp 4, 8 W; Camag, Muttenz, Switzerland). The distance between the UV source and the sample surface was maintained at 90 mm throughout the irradiation process, resulting in a light intensity of approximately 3.5 mW/cm^2^ at the sample surface. All procedures were conducted at 25 °C to prevent thermal denaturation of the collagen, with temperature regulation achieved using a Peltier temperature controller (VPE-35 and VTH1.8K-70S; VICS, Tokyo, Japan). To monitor potential water evaporation during the 120 min irradiation period, sample weights were measured before and after UV exposure using an analytical balance (accuracy: ±0.1 mg). No significant weight loss was observed, indicating minimal evaporation of the water. The sample temperature was monitored using a thermographic camera (ARTCAM-320-THERMO-WOM-16, Artray, Tokyo, Japan), which confirmed that the sample temperature did not exceed 27 °C during the irradiation process.

### 3.3. Preparation of pH Solutions

Eight different pH solutions were prepared for immersion of the gel samples, targeting pH values of 4, 5, 6, 7, 8, 9, and 10. All buffer solutions were made using 0.1 M stock solutions of MES, Tricine, HEPES, CHES, and NaOH. MES buffer was used to prepare a pH 4 solution. A solution of pH 5 was prepared using tricine buffer. For the pH 6 solution, a mixture of 25 mL of MES buffer and 10 mL of NaOH was employed. The pH 7 solution consisted of 25 mL of HEPES buffer and 5 mL of NaOH. The pH 8 solution was prepared by mixing 25 mL of HEPES buffer with 20 mL of NaOH. The pH 9 solution was prepared by combining 25 mL of CHES buffer with 5 mL of NaOH. Finally, a solution with pH 10 was prepared using 25 mL of CHES buffer and 20 mL of NaOH. Each buffer solution was thoroughly mixed, and the pH was verified using a calibrated pH meter to ensure that the target pH values were accurately achieved. Each buffer solution was prepared by dissolving the respective buffer salts in distilled water, followed by adjustment of the target pH using NaOH or HCl. The final pH was determined using pH test papers (Duotest^®^; Macherey-Nagel Co., Ltd., Düren, Germany).

### 3.4. Rheological Characterization (Dynamic Viscoelastic Analysis)

Dynamic viscoelasticity measurements were performed at a controlled temperature of 25 °C on pregel solutions subjected to varying durations of UV treatment (0, 40, and 120 min) to investigate the effect of UV exposure on their viscoelastic properties. The storage and loss moduli were determined using an MCR-101 rotary rheometer (Anton Paar, Graz, Austria) with a plate-plate geometry (25 mm in diameter and a 0.7 mm gap). Measurements were performed at a frequency (ω) of 0.1 rad/s with a strain value of 3%, which was chosen to ensure that the deformations remained within the linear viscoelastic region. The storage modulus (G′) and loss modulus (G″) were recorded as a function of the UV exposure period.

### 3.5. Characterization of pH-Responsive Swelling and Its Reversibility

Hydrogel discs (20 mm in diameter and 2 mm thick) prepared as described in Section 3.2 were subjected to pH-responsive swelling studies. The procedure began with an initial swelling step, in which the hydrogel discs were immersed in ultrapure water for 1 h. After this period, excess surface water was carefully removed using a Kimwipe, and the swollen hydrogels were weighed (W₁). Following the initial swelling, the pre-swollen hydrogels were immersed in pH buffer solutions ranging from 4 to 10 prepared as described in Section 3.3 for 24 h. Excess surface water was removed using a Kimwipe before weighing the hydrogels (W₂). The swelling ratio was then calculated using the following equation: swelling ratio = W₂/W₁. Here, W₁ represents the weight after initial swelling in ultrapure water, and W₂ is the weight after swelling in the pH buffer solution.

The reversibility of the pH response was evaluated using the following procedure. The prepared hydrogels were first immersed in ion-exchanged water for 1 h, after which their weights were measured. Next, the hydrogels were immersed in a pH 4 buffer solution for 3 days and reweighed. Before changing to the next buffer solution, the hydrogels were immersed in distilled water for 3 days. This was followed by immersing them in a pH 10 buffer solution for 3 days and weighing them again after 3 days of immersion in ion-exchanged water. Finally, after another 3 days of immersion in distilled water, the hydrogels were immersed in the pH 4 buffer solution for a second 3 day period and weighed once more. Thus, for the reversibility evaluation, the hydrogels were subjected to solvent exchanges in the order of pH 4, pH 10, and pH 4, with a 3 day distilled water immersion period between each buffer solution change. Similarly, the reversibility was also assessed by changing the solvent in the order of pH 10, pH 4, and pH 10, following the same procedure of distilled water immersion between buffer changes. To evaluate the stability and reversibility of the swelling behavior, measurements were performed twice on similarly adjusted gels and averaged. All of the measurements were performed at room temperature (25 ± 1 °C) using an analytical balance with an accuracy of 0.1.

### 3.6. Mechanical Properties at Different pH Values

The elastic modulus of the hydrogels at various pH values was determined using a rheometer (Creep Meter RE2-33005B, Yamaden, Japan) at room temperature (20 °C). For elasticity measurements, hydrogel samples were prepared by injecting 0.125 g of riboflavin-collagen solution into each well of a silicone mold with a flat bottom (well dimensions: 1 cm × 1 cm). UV irradiation was applied using the same equipment described in Section 3.2 to obtain collagen gels within the mold. The gels were prepared with UV irradiation times of 40 min and 120 min. Following gel formation, 0.125 g of the pH solutions prepared in Section 3.3 was added to each mold well containing the gel. The samples were then left to equilibrate for 1 d to ensure complete swelling. Push-in tests were performed on the gels while they remained in the mold cells. The measurement conditions were as follows: strain rate of 30%; measurement speed of 1 mm/s; contact area diameter of 3 mm; fixture diameter of 3 mm; and applied temperature of 24 °C. Stress–strain curves were obtained for 12 collagen gel samples. Young’s modulus was calculated from the slope of the linear region in each stress–strain curve.

### 3.7. Fibroblast Proliferation Assay

Fibroblast proliferation on collagen hydrogels was evaluated using visual counting with a phase-contrast microscope. Collagen gels were prepared as described in Section 3.2, with UV irradiation times of 40 min, 60 min, and 90 min for the proliferation test. NIH-3T3 fibroblasts (ATCC, Manassas, VA, USA) were cultured in Dulbecco’s modified Eagle’s medium (DMEM; Gibco, Thermo Fisher Scientific, Waltham, MA, USA) supplemented with 10% fetal bovine serum (FBS; Gibco) and 1% penicillin-streptomycin (Gibco). Cells were maintained at 37 °C in a humidified atmosphere containing 5% CO₂. For the proliferation assay, fibroblasts were trypsinized, counted, and seeded onto the freshly UV-irradiated collagen gels at a density of 1.8 × 10⁴ cells/cm^2^. The seeded gels were placed in 5.2 mm diameter glass petri dishes and incubated in complete DMEM at 37 °C in a 5% CO₂ incubator. Cell proliferation was monitored using a phase-contrast microscope (Leica DMI3000 B; Leica Microsystems, Wetzlar, Germany) equipped with a 10× objective lens. Observations were made at two time points: immediately after seeding (0 h) and 48 h (2 days) post seeding. At each time point, three random fields of view per gel sample were photographed using a digital camera (Nikon D50, Nikon Corporation, Tokyo, Japan). Cell numbers were manually counted using ImageJ software (version ver.1.54h, National Institutes of Health, Bethesda, MD, USA).

## 4. Conclusions

In this study, we successfully developed pH-responsive collagen hydrogels using UV irradiation with riboflavin as the photosensitizer. Our key findings revealed that these hydrogels exhibited significant pH-dependent swelling behavior, with maximum swelling at a pH of four and minimal swelling above a pH of six. The partial reversibility of the pH response was observed over multiple pH cycles, demonstrating the potential of the hydrogels for repeated use in dynamic environments. The elastic modulus of the hydrogels showed a 10 fold increase from a pH of 6 to 10, indicating pH-dependent mechanical properties. Fibroblast proliferation assays confirmed the biocompatibility of the hydrogels, with cell growth positively correlating with the UV exposure time.

The demonstrated pH-responsive behavior and biocompatibility of these UV-crosslinked collagen hydrogels present numerous potential applications, particularly in the fields of wound healing dressings which can adapt to the changing pH environment of the wound bed and targeted drug delivery systems capable of selective release. The release of therapeutic agents is in response to specific pH conditions, modulation of the mechanical properties in tissue engineering scaffolds in response to pH changes, monitoring of pH changes in vivo or in various industrial applications, and adjustment of the effects in cosmetic formulations based on the pH of the skin or hair. Recent research on pH-responsive hydrogels has demonstrated the possibility of achieving complex 3D shape transformations through the precise control of internal stresses in hydrogels [82,83]. Exploring similar concepts in biocompatible materials, such as collagen, could open new avenues for biomedical applications. These potential applications highlight the versatility and promise of our pH-responsive collagen hydrogel, warranting further investigation and development.

Future research should focus on enhancing the reversibility of the pH response, particularly after exposure to high pH levels, and investigating the incorporation of stabilizing agents to improve the long-term stability under physiological conditions. As we continue to develop and refine these materials, we anticipate that UV-crosslinked pH-responsive collagen hydrogels will play a significant role in advancing the field of smart biomaterials for medical and biotechnological applications. The outcomes of this study are expected to make substantial contributions to various scientific and industrial sectors, paving the way for innovative solutions in healthcare, drug delivery, and other fields.

## Data Availability

Data are contained within the article.

## Supplementary Materials

The following supporting information can be downloaded at: https://www.mdpi.com/article/10.3390/ijms251910439/s1.
