# Supplementary material for: pH-Responsive Collagen Hydrogels Prepared by UV Irradiation in the Presence of Riboflavin
Source: Int J Mol Sci. 2024 Sep 27;25(19):10439. doi: 10.3390/ijms251910439 (PMC11476811; doi:10.3390/ijms251910439)
Supplement: Supplementary file 1 [file ijms-25-10439-s001.zip › ijms-3197303-supplementary.pdf]

Supplementary Materials

pH-Responsive Collagen Hydrogels Prepared by UV Irradiation in the Presence of Riboflavin

Shoki Setoyama [1], Ryota Haraguchi [1], Shigehisa Aoki [2], Yushi Oishi [1] and Takayuki Narita [1,*]

Table of Contents

1. SUPPLEMENTAL FIGURE

Figure S1: Microscopic images of fibroblasts on UV cross-linked collagen hydrogels immediately after seeding.

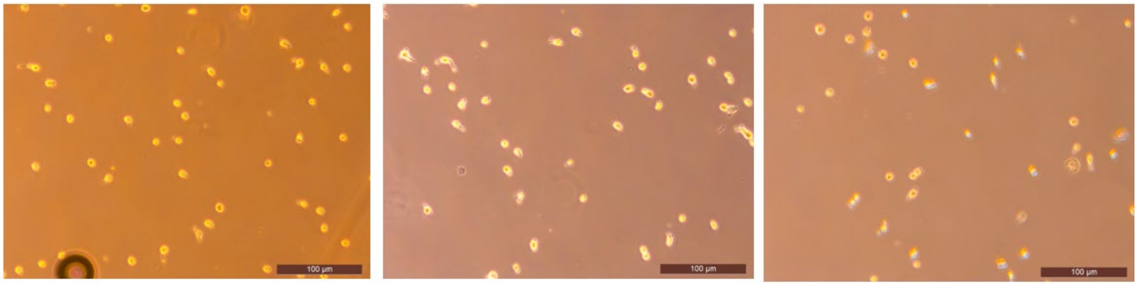

(a) (b) (c)

Figure S1: Microscopic images of fibroblasts on UV cross-linked collagen hydrogels immediately after seeding. Hydrogels were prepared with UV irradiation times of (a) 40 min, (b) 60 min, and (c) 90 min. Scale bar represents 100 µm.
